# Towards Automated Measurement of As-Built Components Using Computer Vision

**DOI:** 10.3390/s23167110

**Published:** 2023-08-11

**Authors:** Husein Perez, Joseph H. M. Tah

**Affiliations:** Oxford Institute for Sustainable Development, School of the Built Environment, Oxford Brookes University, Oxford OX3 0BP, UK; jtah@brookes.ac.uk

**Keywords:** machine learning, computer vision, automated measurement

## Abstract

Regular inspections during construction work ensure that the completed work aligns with the plans and specifications and that it is within the planned time and budget. This requires frequent physical site observations to independently measure and verify the completion percentage of the construction progress performed over periods of time. The current computer vision techniques for measuring as-built elements predominantly employ three-dimensional laser scanning or three-dimensional photogrammetry modeling to ascertain the geometric properties of as-built elements on construction sites. Both techniques require data acquisition from several positions and angles to generate sufficient information about the element’s coordinates, making the deployment of these techniques on dynamic construction project sites challenging. This paper proposes a pipeline for automating the measurement of as-built components using artificial intelligence and computer vision techniques. The pipeline requires a single image obtained with a stereo camera system to measure the sizes of selected objects or as-built components. The results in this work were demonstrated by measuring the sizes of concrete walls and columns. The novelty of this work is attributed to the use of a single image and a single target for developing a fully automated computer vision-based method for measuring any given object. The proposed solution is suitable for use in measuring the sizes of as-built components in built assets. It has the potential to be further developed and integrated with building information modelling applications for use on construction projects for progress monitoring.

## 1. Introduction

The accurate and up-to-date measurement of as-built components is essential for the design, construction, operation, and maintenance of as-built assets. Additionally, the measurement of as-built components is an essential part of construction project management functions, such as cost and schedule controls, financial reporting, claims, and productivity measurement. It is, therefore, considered to be one of the most crucial, yet challenging tasks facing site managers. Most current approaches are still predominately manual, time-consuming, and error-prone. Site managers normally spend a significant amount of time measuring, recording, and analysing as-built information [1,2,3]. The lack of accurate and up-to-date as-built information due to laborious and manual data collection practices could lead to increased costs, delays, and poor project performance, which in turn, could reduce the ability to detect or manage the variability and uncertainty inherent in the project’s activities [4,5,6].

In recent years, however, the construction industry has been exploring various emerging technologies to support the visual inspection and progress monitoring of construction work [7]. The on-site application of these technologies has indeed demonstrated significant potential for digitising and automating the capturing, measuring, and reporting updates of the as-built components and project information [6,8].

One notable example of these technological tools for automating the measurement of as-built components is the use of computer vision (CV). CV is a digitisation process used for determining project progress that combines computer science, architecture, construction engineering, and management disciplines. It takes visual media, such as photos, videos, or scans as inputs and produces decisions or other forms of representation as outputs [9].

The two most popular CV-based techniques for measuring the as-built components are laser-based scanning, and imaging-based photogrammetry [4,8,10,11]. Three-dimensional (3D) laser scanning is used to generate 3D point clouds that are processed to enable the estimation of sizes and quantities of as-built components [12]. The imaging-based approach, on the other hand, emulates human visualisation to extract three-dimensional (3D) geometrical information of objects from two-dimensional (2D) inputs [13,14,15].

A fully automated CV-based method for measuring as-built components consists of four main sub-processes; data acquisition, information retrieval and processing, measurement estimation, and producing valuable output [16,17,18,19]. The sub-processes involve different techniques to achieve the desired outputs with their own benefits and limitations [8,20].

Despite some studies having made significant strides in automating CV-based methods for as-built component measurements, at present, there are still no applications that are fully automated. This is due to two main reasons. Firstly, the technologies involved are still emerging and undergoing experimentation with only a few functional demonstrations available [21]. Secondly, existing studies do not typically address the four stages together but focus on the individual stages, such as 3D point cloud generation in data acquisition [22,23], and feature recognition in information retrieval and processing [24,25,26].

This study aims to address this research gap by developing a pipeline for a fully automated as-built component measurement approach using CV-based methods. The proposed pipeline can run in real-time and is intended to estimate the size of as-built components of built assets. The pipeline employs stereo camera techniques for data acquisition, machine learning, object detection, instance segmentation for information retrieval and the processing of as-built elements, Green’s theorem [27] for the measurement estimation of the size of the object(s) under consideration, and visualisation of the output as labelled images. To demonstrate the work, s neural network model was trained on concrete walls and columns, but the same principles can be extended to cover other types of as-built components.

The rest of this paper is structured as follows: Section 2 provides a literature review of related previous studies, highlighting the existing research and theories relevant to the subject matter. Section 3 focuses on the instrumentation and materials used in this study, outlining the experimental setup and tools employed for data collection. We delve into the methodology of geometric estimation, explaining the mathematical models and algorithms utilised to estimate the geometric properties of the as-built components of interest. Section 4 presents the results obtained from the experiments, including a detailed analysis and discussion. Finally, Section 5 summarises the key findings and conclusions drawn from this study, highlighting the implications, significance, and limitations of this research.

## 2. Literature Review

The measurement of as-built components is crucial in the design, construction, operation, and maintenance of built assets. It plays a vital role in continuously monitoring and periodically updating the actual work conducted on a construction site, comparing it with the as-planned or anticipated progress [28,29,30]. Identifying variations between the planned and actual progress is essential for schedule updating [31]. The most common CV-based method for undertaking spatial measurements of the actual work on construction sites is 3D laser scanning. During the process, the construction site is scanned from different angles and locations at different times to generate spatial data, which can then be used to estimate the quantities of work performed within the time interval considered between two successive scans. A 3D laser scanning yields data in the form of 3D points, known as “point clouds”, which are later displayed as images that can be viewed from different perspectives using specialised software systems [32,33]. Many researchers have proposed and demonstrated that the technology can be used to obtain 3D data on the actual progress of a project efficiently [31,34,35,36,37].

However, this method has certain limitations as 3D data can be obtained only on the as-built components that are located within the laser scanner’s range and field of view. Secondly, even components that are physically within the range of the scanner may still be blocked from view by various pieces of equipment and other obstacles located around the construction site, resulting in an incomplete 3D data set obtained on a construction site. To overcome this problem, researchers have proposed UAV-based 3D laser scanning methods [38,39,40,41,42,43,44,45,46]. The authors argued that this approach can provide visual and detailed progress information with good area coverage and views from human-inaccessible angles. UAV-based data acquisition, however, requires careful operation handling as it can pose potential safety hazards and cause distractions to workers on-site. UAVs also require accurate path planning to avoid obstruction, which in the case of any sudden rotational motion or sharp angular movements can result in motion blur. They can also be affected by wind speeds and other environmental anomalies.

In addition to issues related to the acquisition approach, there are other limitations associated with 3D laser scanning. This includes the time required to perform a single scan, and the number of scan positions necessary to acquire accurate information. The technique is also costly, technically intricate, and requires skilled experts to capture and model the whole project. Moreover, the collected 3D point cloud also requires extensive time and computational resources to process data and produce meaningful interpretations, which may not be adequate for use in complex project sites to generate real-time updates [20]. The incomplete or partially occluded patches in a 3D point cloud will also incur technical challenges during the registration of multiple point clouds [23,47,48,49].

On the other hand, the availability of high-quality and precise still image cameras has advanced 3D modelling from photo images [50]. As a result, an image-based scanning method called photogrammetry has been proposed as an alternative to 3D laser scanning [51]. With photogrammetry, the geometrical properties of an object on site are generated from its photo image. The technique, however, requires strategically placing many targets on the object(s) being photographed to identify the object’s coordinates, and several photos of the object are then taken from different positions and angles to generate sufficient information on object coordinates [52]. The use of image-based scanning may also incur other practical limitations, particularly when extracting geometrical properties of surfaces with little texture or poor definition [53]. Additionally, a recent study has also shown that the accuracy of the model generated from the image-based reconstruction is less than the laser scanner and becomes even less accurate as the length of the element increases. According to the study, the process of reconstructing a 3D model from an image dataset remains reliant on human intervention at various steps to improve the output quality [54].

Compared to existing photogrammetry techniques that require the placement of many targets on the object and several photos taken from different positions and angles to generate sufficient geometrical information, the proposed method requires a single image obtained from a stereo camera system and a single target to extract the information about the object’s coordinates. Additionally, unlike 3D laser-based scanning, which also requires the construction site to be scanned from different locations and generates computationally extensive 3D point clouds, the proposed pipeline is capable of generating real-time updates of as-built components on construction sites.

## 3. Instrumentation

**Figure 1:**
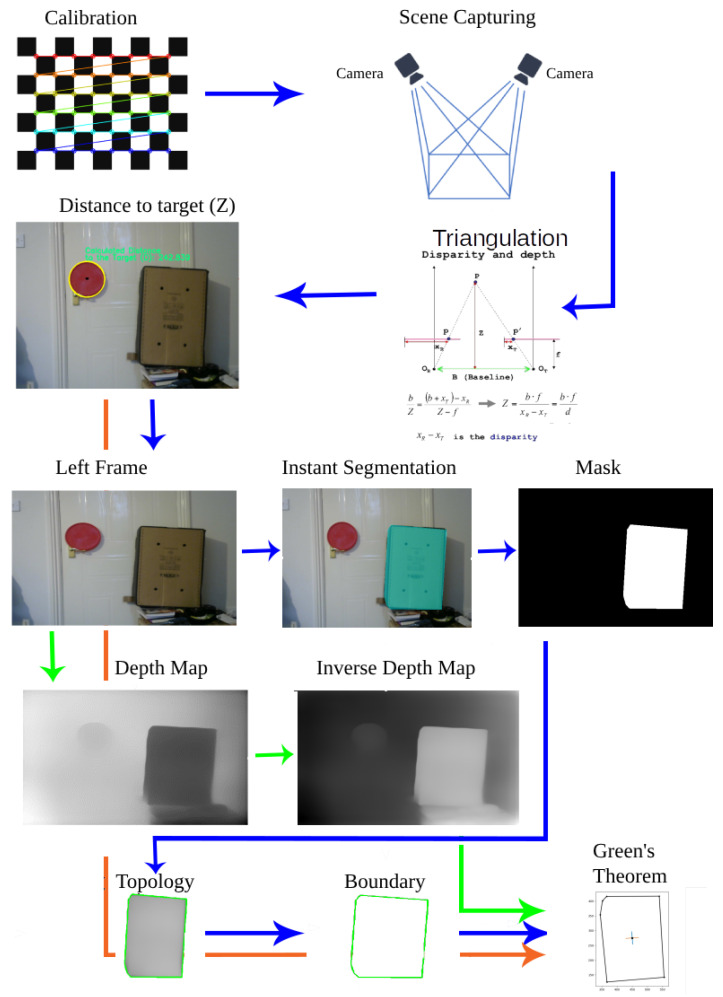
The proposed pipeline showing all sub-stages: stereo camera calibration, scene capturing, object segmentation, generation of the absolute depth map, extraction of the boundary of the object, and finally area estimation.

In this section, we discuss the pipeline for the full CV-based method, which was developed for measuring as-built components. To demonstrate the results, images containing concrete walls and columns captured from buildings at Oxford Brookes University, Headington campus, was used to apply the pipeline to estimate the sizes and areas of concrete elements. The pipeline, which is depicted in Figure 1, can run in real-time, and it comprises seven steps: camera calibration, scene capturing, calculating the distance to a point in the scene, instance segmentation, depth map generation, estimating world coordinates, and finally calculating the area of the object of interest. This section will be divided into four subsections: data acquisition; information retrieval and processing; as-built component measurements; and finally visualisation of the output.

### 3.1. Initialisation

**Camera calibration:** a stereo camera system was used to capture the scene containing the object of interest. It is important for the camera system to be accurately calibrated. The calibration is a one-time process used for determining the intrinsic (principle point, distortion parameters, and focal length) and the extrinsic parameters (rotation and translation) of each camera and the relative poses between them. These parameter sets are essential for attaining 3D information given a set of 2D coordinates of corresponding image points [55]. The process of recovering the third missing dimension is an ill-posed problem and is known in image geometry applications as depth estimation [56,57].

There are many different techniques used for approximating the intrinsic and extrinsic parameters for a specific camera model. The most common one is Zhang’s method [58], (the one adopted in this work) and the direct linear transformation (DLT) [59].

Zhang’s method uses multiple views of a 3D pattern of a known structure but an unknown position and orientation in space. It is a flexible technique for camera calibration and well-suited for use without specialised knowledge of 3D geometry or computer vision. The technique only requires the camera to observe a planar pattern shown at a few (at least two) different orientations. During the calibration process, both the camera and the planar pattern can be freely moved, but motion does not need to be known [58,60].

DLT, on the other hand, is a mathematical approach that aims to solve the problem of determining the pinhole camera parameters from at least six correspondences between 2D image points and 3D world points. A camera model maps each point of the 3D world to a point of the 2D image through a projection operation. The pinhole camera model makes the assumption that the aperture size of the camera is small so that it can be considered a point. Thus, the ray of light has to pass across a single point and the camera centre; there are no lenses, no distortion, and there is an infinite depth of field [59,61].

In their simplest form, the intrinsic parameters can be represented by a 3×3 matrix called the camera matrix, denoted by the letter *K*, as presented below:

(1) $$K=\left[\begin{array}{ccc} f_{x} & 0 & c_{x}\\ 0 & f_{y} & c_{y}\\ 0 & 0 & 1 \end{array}\right]$$

where $f_{x}$, $f_{y}$ are the lengths of the focal point (in pixels), which is the distance from the centre of the lens to the principal points of the lens. $c_{x}$, $c_{y}$ are the principal points which are the points on the image where a ray of light travelling perpendicular to the image plane passes through the focal point of the lens and intersects with the camera’s sensor.

### 3.2. Data Acquisition

**Figure 2:**
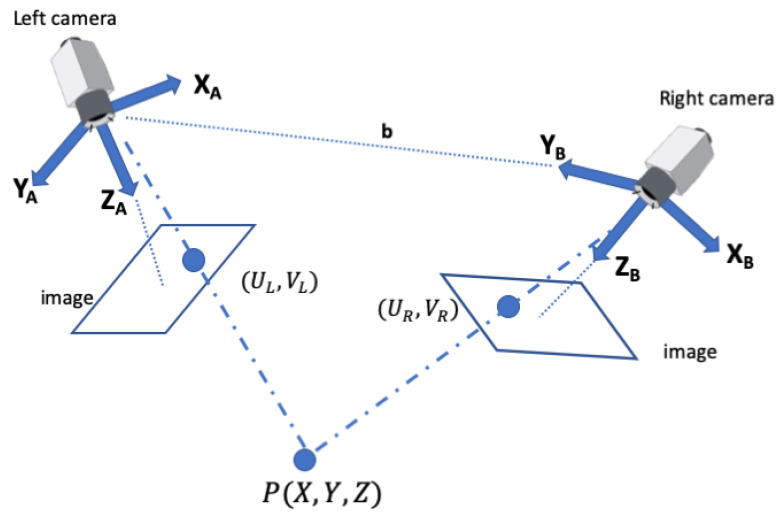
Typical stereo vision system: **b** represents the distance between the principal points, $\left(U_{L},V_{L}\right)$, $\left(U_{R},V_{R}\right)$ are the 2D projections of the real-world point P(X,Y,Z).

**Scene capturing:** A stereo vision system, also known as binocular stereo vision, is a machine vision technique that uses exactly two cameras to capture a scene from two viewpoints. The two cameras are separated by a short distance known as the baseline *b* and are mounted almost parallel to one another. The principle of stereo vision is similar to that of the 3D perception of the human eyes. It can provide a 3D perception with real-time depth measurements based on the triangulation of rays from the two viewpoints (see Figure 2).

*b* is the baseline, *f* is the focal length of the camera, and $u_{L}$ and $u_{R}$ are the projections of the real-world point *P* in an image acquired by the left and right cameras. $X_{A}$ and $Z_{A}$ are the X-axis and the optical axis of the left camera, respectively, whereas $X_{B}$ and $Z_{B}$ are the X-axis and the optical axis of the right camera, respectively. *P* is a real-world point defined by the coordinates *X*, *Y*, and *Z* [62].

**Calculating the distance to the target (*Z*)**: In order to calculate the depth of information *Z*, which is the distance to the real-world point *P*, we first calculate the disparity *D*, which is the horizontal shift in position between two corresponding points projected on the image plane in the stereo vision system. In this approach, a red circular target was used to calculate the horizontal disparity between the centres of the circles appearing in the left and right frames. It is important that both the target and the object of interest are visible in the two frames, and that both the left and the right frames have at least a 30% overlap. The calculations of the depth and disparity values are shown in (2) and (3).

(2) $$D=u_{L}-u_{R}$$

(3) Z=f∗b/D

where *b* is the baseline, *f* is the focal length of the camera obtained from (1), $u_{L}$ and $u_{R}$ are the projections of the real-world point *P* in an image acquired by the left and right cameras [63].

**Figure 3:**
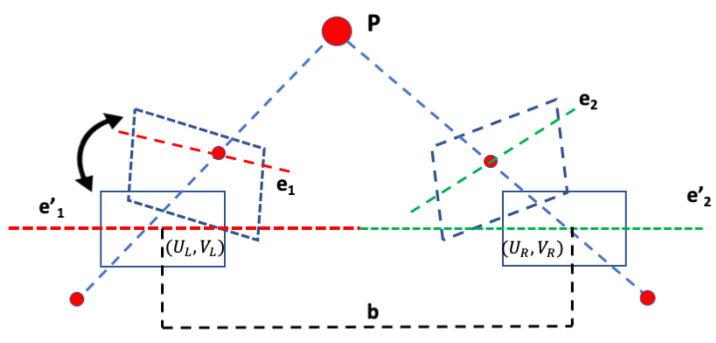
Illustration of image rectification. Epipolar lines $e_{1}$, $e_{2}$ are projected on a common line (dashed red–green). The distance between principle points $\left(U_{l},V_{l}\right)$ and $(U_{R},V_{R}$) along the common line is the baseline **b** [65].

When capturing a scene from two distinct viewpoints using a stereo camera system, the left and right frames are not lined up perfectly, and when the cameras rotate or move forward or backward, the pixels will also move accordingly. This makes matching the corresponding pixels in each frame a very challenging task. To simplify the subsequent stereo correspondence problem, a process called rectification is applied first (see Figure 3). Stereo rectification is the determination of two image transformations (or *homographies*) that map corresponding points on the two images and projections of the same point in the 3D space onto the same horizontal line in the transformed images [64,65].

The target can be placed anywhere in the scene and only needs to be visible by both cameras. Placing the target closer or on the object of interest would, however, improve accuracy. A target is any artefact object that can be distinguished from the surroundings, either by shape or colour. A red circular object was used as a target to facilitate image processing techniques to detect the (red) colour and determine the circumference of the target in both left and right frames. The circular shape allows us to easily obtain the centre of the disk in both frames. Therefore, the horizontal displacement (the disparity *D*) between the two centres in the left and right frame is the difference between the *x* components of the target’s centre point ($x_{L}-x_{R}$). To calculate the depth (i.e., the distance to the target (*Z*)) the triangulation method was applied to estimate the absolute distance to the target using (2) and (3).

**Figure 4:**
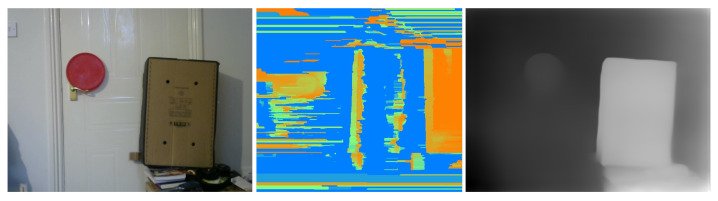
Depth maps: original image (**left**), deteriorated sparse depth map (**middle**), CNN-based dense depth map (**right**).

**Depth map generation:** The depth estimation to all corresponding points projected on the image plane in the stereo vision system using the triangulation method will, inevitably, generate depth maps that are, in most cases, rough and sparse wherever matching between corresponding pixels fails [66]. Meanwhile, with the rapid development of deep/convolutional neural networks (CNNs), monocular depth estimation based on deep learning has been widely studied recently, showing promising accuracy. These CNN-based methods are able to generate dense depth maps from single images where the depth at every pixel in the image is estimated by the neural network in an end-to-end manner [67,68,69] (see Figure 4). With CNN-based methods, the estimation of the absolute depth (i.e., depth from the object to the camera) directly from a single image can be ambiguous in scale; for example, an object may appear to be the same as another identically shaped but smaller object at a nearer distance [70]. The relative depth, on the other hand, which is the ratio between the depths of two points in an image, is scale-invariant. This principle also applies to humans since it is easier to choose the nearer between two points than to estimate the absolute depth of each point; therefore, relative depths are easier to estimate than ordinary (absolute) depths [70]. The adopted monocular depth estimation in this work [71] generates a relatively dense depth map of each pixel in a single image with values between 0 and 1.0, where pixels with higher values are closer to the camera, and pixels with small values are further from the camera. By inverting this dense map, it is possible to assign small relative distances to closer pixels to the camera and higher relative distances to the furthest pixels from the camera.

**Figure 5:**
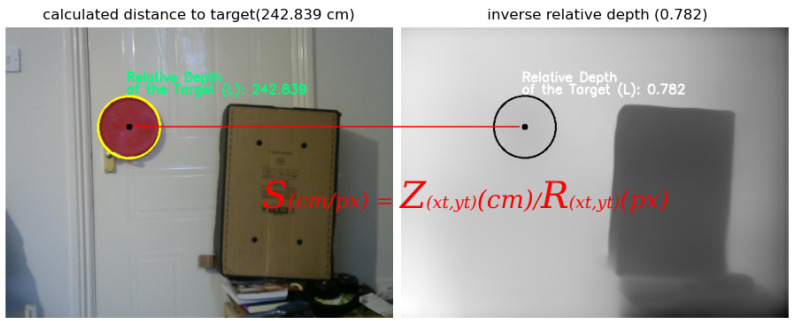
*S* is the ratio of the absolute distance *Z* in the left image to the relative distance *R* from the depth map in the right image.

Next, the (absolute) distance of the target obtained from (2) and (3) is used to compute a scalar *S*, such that:

(4) $$S_{cm/px}=\frac{Z_{xt,yt}\left(cm\right)}{R_{xt,yt}\left(px\right)}$$

where $Z_{xt,yt}$ is the (absolute) distance to the centre of the target in cm, and $R_{xt,yt}$ is the (relative) distance to the centre of the target in pixels (see Figure 5).

In this scenario, the scale S=242.839/0.782=342.857. By multiplying the scalar *S* with every entry in the relatively dense depth map, it is possible to generate the absolute depth map. To illustrate this, suppose a point $p_{i}$ is a pixel anywhere in the image, with relative distance equal to the one at the centre of the target $R_{xi,yj}=0.782$, i.e., both the point $p_{i}$ and the centre of the target are the same distance from the camera, thus, the absolute distance at $p_{i}=0.782\times 342.857=242.389$ cm, which is the same absolute distance of the target from the camera. Similarly, if $p_{j}$ is a pixel with relative distance $R_{xi,yj}=0.384$, which is closer to the camera than the target, then the real distance of that point is $p_{j}=0.384\times 342.857=130$ cm.

### 3.3. Information Retrieval and Processing

**Figure 6:**
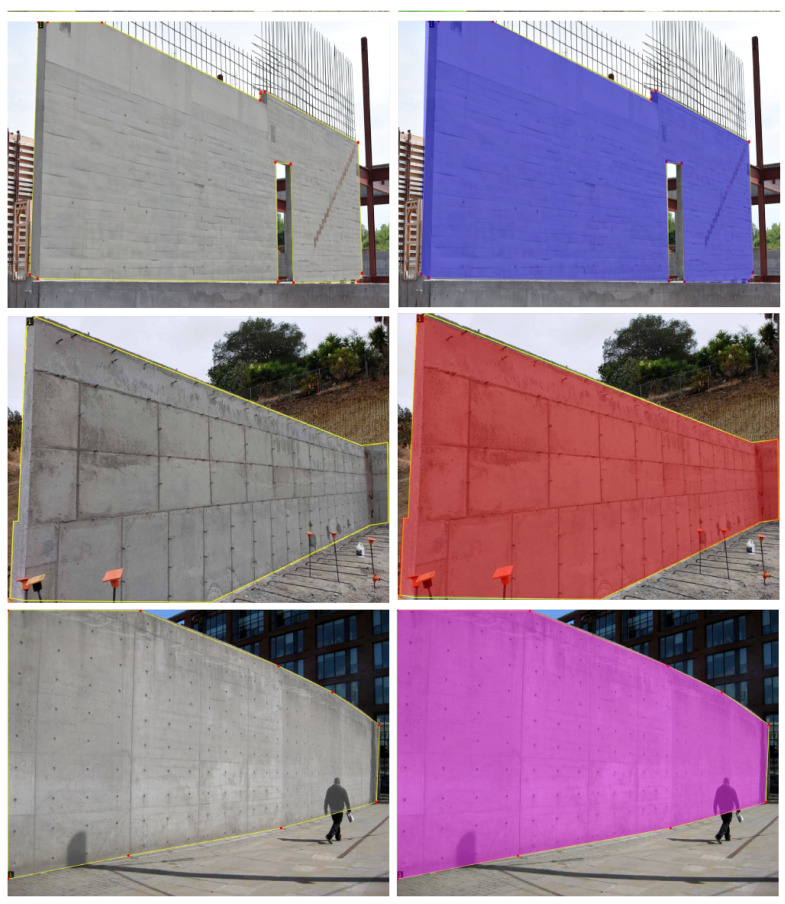
Sample of images with concrete structures used to train the neural network model.

**Instance segmentation:** Now, the attention is turned to information retrieval and processing using object detection. Since the current implementation is devoted to measuring concrete structures (concrete columns and walls) only, a neural network model [72] was trained to extract (segment) those objects from a given image. A sample of the dataset used for training the model is presented in Figure 6).

Object detection and segmentation is the process of identifying the presence of an object in the image. It associates every pixel of that object with a single class label, e.g., a person, box, car, and so on [73]. For every class, the neural network applies a unique colour mask over all the pixels of that object. There are two types of object segmentation: (1) semantic, where the neural network treats multiple objects of the same class as a single entity, and (2) instance segmentation, which, in contrast to semantic, treats multiple objects of the same class as distinct individual instances [73].

**Figure 7:**
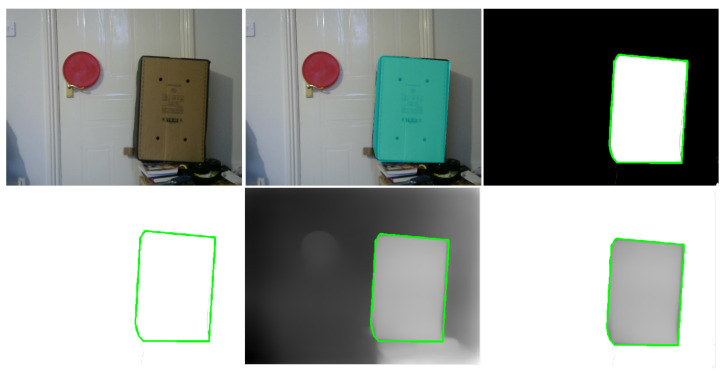
Extraction of the object boundary using the left frame: object detection, mask generation, separation of boundary points from the depth map.

Figure 7 demonstrates the process of using object segmentation in an image to extract the corresponding pixels with absolute depth values of that object from the dense depth map.

First, a single image (left frame) is passed through the neural network for instance segmentation (Figure 7, top left). The model generates a colour mask over the object of interest and assigns all pixels related to the object with a single label (Figure 7, top middle). The corresponding masks of each object, which are saved separately, are used to extract the boundary of that object (Figure 7, top right).

The extracted boundary of the object (Figure 7, bottom left) is projected on the dense depth map containing the computed absolute depths (Figure 7, bottom middle) to separate only those pixels of the object of interest (Figure 7, bottom right). The mathematical formulation of this process is presented in the next section.

### 3.4. As-Built Component Measurement

**Estimate world coordinates.** The next step of the proposed pipeline is to compute the real-world coordinates of the object and estimate the area of the object. The conversion to a real-world coordinate system (in cm) from the image-coordinate system (in pixels) is governed by the following equations:

(5) $$X_{w}=\left(x-c_{x}\right)*Z_{xi,yj}/f_{x}$$

(6) $$Y_{w}=\left(y-c_{y}\right)*Z_{xi,yj}/f_{y}$$

$X_{w}$, $Y_{w}$ are the computed two-dimensional real-world coordinates of each pixel in the object. *x*, *y* are the coordinates of each pixel in the object, $c_{x}$, $c_{y}$ are the principal coordinates of the camera, which are estimated during the calibration, $f_{x}$, $f_{y}$ are the lengths of the focal point (in pixels) also found from the camera matrix (*K*), and $Z_{xi,yj}$ is the absolute distance at that pixel. The code in Algorithm 1 illustrates the procedure of obtaining the real-world coordinates of the object of interest.

**Calculating the area of the object of interest:** Lastly, Green’s theorem [27] was applied to calculate the area of the two-dimensional irregular region, i.e., the closure $\bar{D}$, which is enclosed by the boundary ∂D and denoted in Algorithm 1 as BV.

### 3.5. Visualisation of the Output

The proposed pipeline, which was entirely developed in Python, can produce an output of any text or graphical format. This makes the integration of such an output with any BIM model an easy task. Full demonstrations of the output samples are presented in Figures  10, 11 and 13.
**Algorithm 1:** Real-world coordinates.
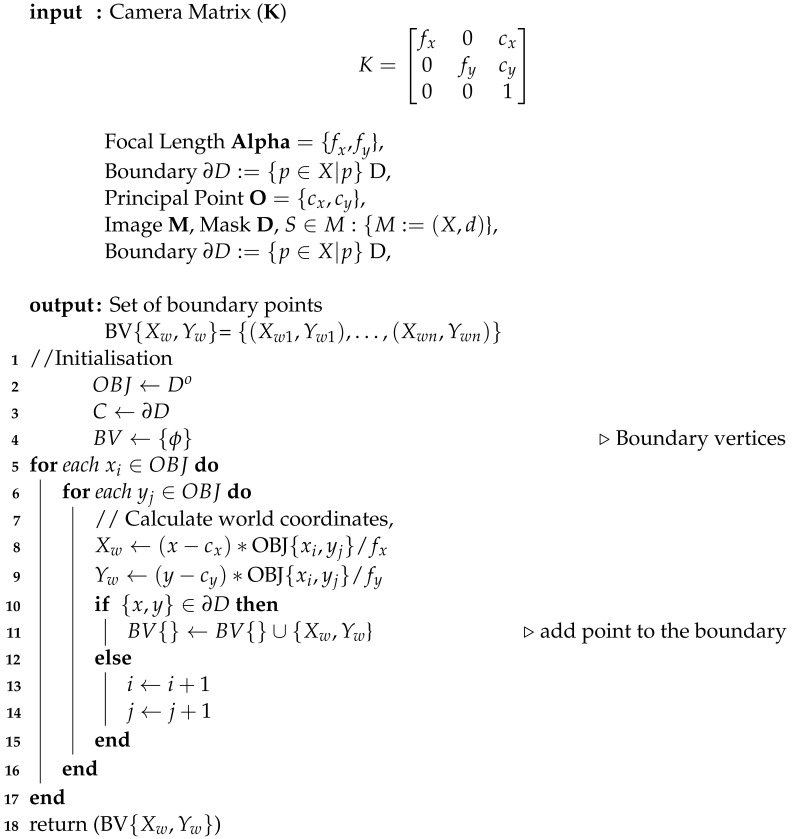


## 4. Results and Discussion

A discussion of the results through two examples follows: one for measuring a concrete column and the other for measuring a concrete wall at an Oxford Brookes University building. The building is a new addition to Oxford Brookes University and is mainly built from concrete components.

**Figure 8:**
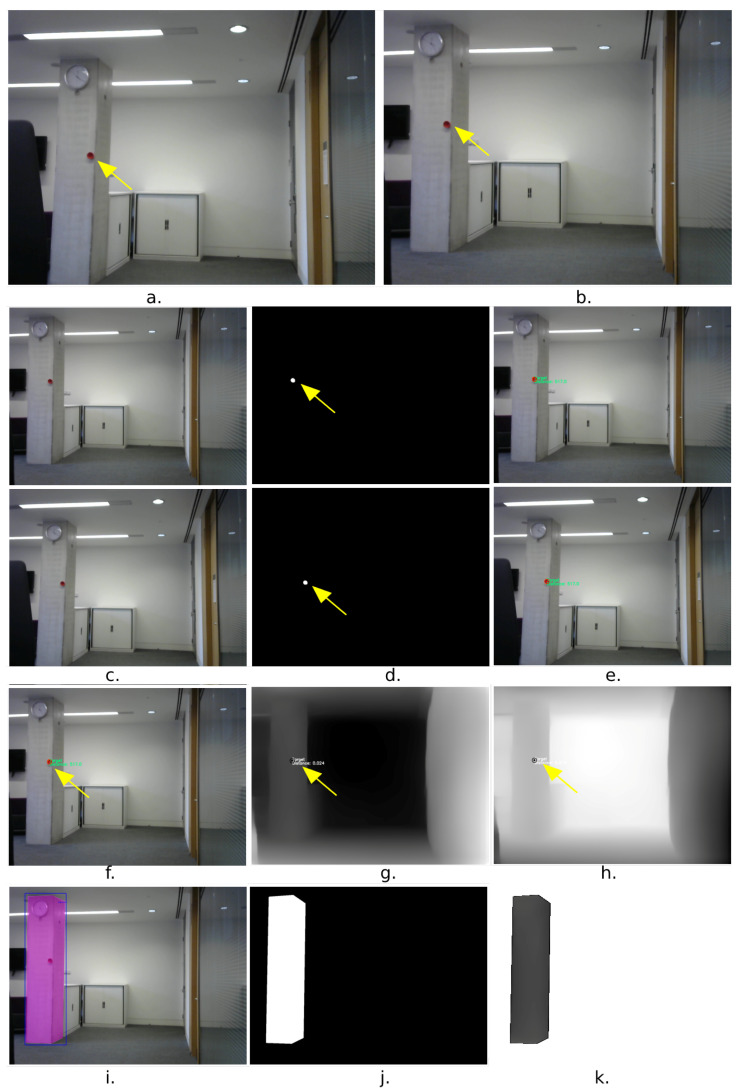
Experiment 1: estimating the area of a concrete column: in the first row scene capture (**a**,**b**), in the second are third row depth estimation to target in the left and right frames respectively (**c**–**e**), in the fourth row, the depth map (**g**), and corresponding inverse (**h**), using the left frame (**f**). In the last row, object segmentation (**i**), masking (**j**), and object extraction (**k**).

In the first experiment, a stereo camera system with a baseline (b=25 cm) was used to capture the left and right frames of the scene. The stereo camera system was placed at a distance of 5.2 m away from the column. The distance and the angle of the acquisition were chosen randomly, allowing the whole object (a concrete column) to appear completely in both frames. A target (red circular disk) was placed on the object at the same level of the stereo system, where it was also visible in both cameras (Figure 8a,b).

**Figure 9:**
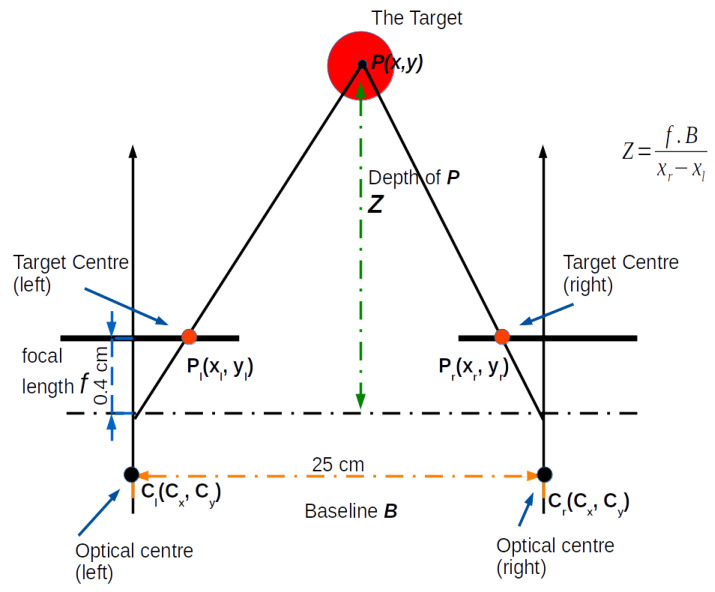
Depth from disparity: P(x,y) is the real-world coordinates of the target centre, *Z* is the calculated distance (Depth) to the centre of the target. $P_{l}$ and $P_{r}$ represent the coordinates of the centre of the target in the left and right images, respectively. $C_{l}$ and $C_{r}$ represent the principal points of the left and right cameras estimated during the calibration stage.

Once the scene was captured, a (red) colour filter was applied to allocate the target in both frames and calculate the coordinates of the centre of the circle in the left frame $(x_{l}$, $y_{l})$ (Figure 8d-top row) and in the right frame (Figure 8d-bottom row) $\left(x_{r},y_{r}\right)$, respectively. Next, using the principles of triangulation demonstrated in Figure 9, the depth $Z_{xt,yt}$ (e.g., distance to the centre of the target) was calculated using the following formula:

(7) $$Z_{xt,yt}=\frac{f\cdot B}{X_{r}-X_{l}}$$

where *f* is the horizontal focal length (in pixels), *B* is the baseline, and $x_{r}-x_{l}$ is the horizontal disparity. In this experiment, the calculated depth $Z_{xt,yt}$ to the centre of the target was 5.17 m and the result is displayed in the left frame (Figure 8e-top row) and the right frame (Figure 8e-bottom row), respectively.

The left frame (Figure 8f) was then passed through the CNN-based monocular depth estimation model to generate the inverse dense depth map containing the relative depths of the scene (Figure 8g). The dense depth map was then inverted again so that a small relative depth indicates the closer points and the larger values refer to further points (Figure 8h).

To calculate the scalar *S* for this experiment, both $Z_{xt,yt}=517$ cm and $R_{xt,yt}=0.976$ were used, referring to the relative depth values obtained from the inverted depth map (Figure 8h) at points $x_{t},y_{t}$, i.e., the target centres.

The scalar *S* is case-dependant, i.e., it varies depending on the position of the target in that scene. For this case, the scalar *S* is calculated as follows:

(8) $$S=\frac{D_{cm}}{R_{px}}=\frac{512}{0.976}=529.713\;\mathrm{cm}/\mathrm{px}.$$

Now, the inverted depth map shown in Figure 8h was multiplied by the scalar *S* to generate a dense map with absolute depths.

To extract the object (the concrete column) from the scene, the left frame was passed through the trained module for object detection and instance segmentation as shown in Figure 8i. The output mask is shown in Figure 8j; corresponding to the detected object, i.e., the concrete column, it is projected on the dense map with the absolute depth values, which were generated in the previous step to extract only those segmented pixels related to the concrete column. Finally, the code in Algorithm 1 was applied to split the vertices belonging to the boundary (BV) and then Green’s theorem was used to estimate the area of the concrete column.

**Figure 10:**
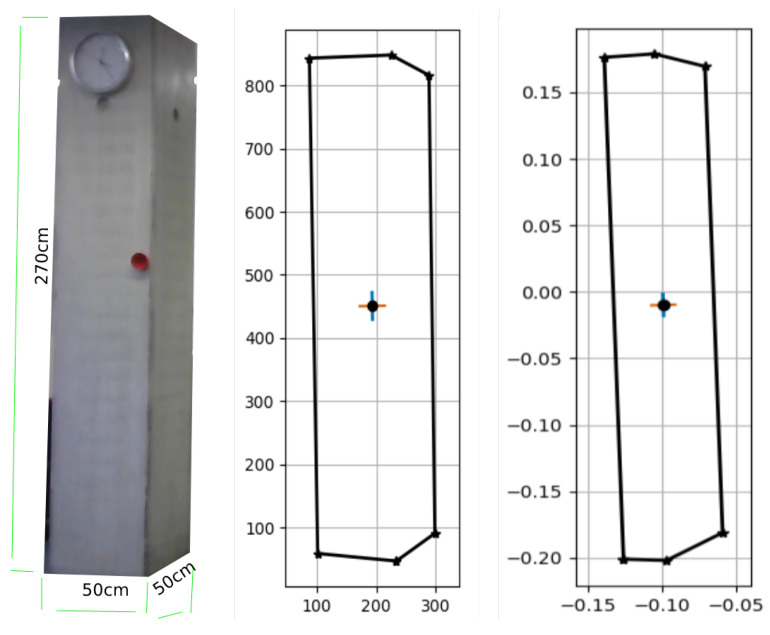
Real-world reconstruction of the concrete column: (**left**) the actual column, (**middle**) the pixel coordinates, and (**right**) real-world reconstruction.

**Figure 11:**
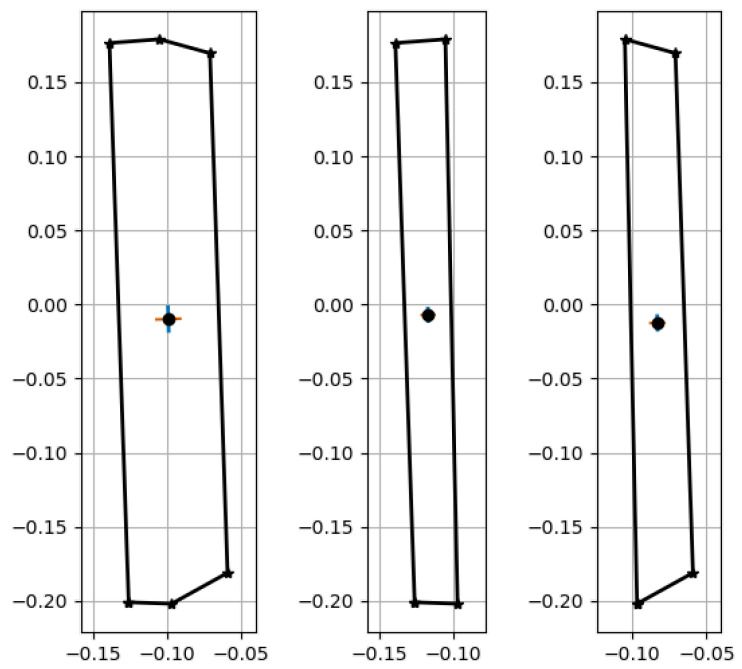
Reconstructed concrete column: the **left** is the total surface area, the **middle** is the front face area, and the **right** is the side face area.

The actual surface areas of the column are shown in Figure 10(left), as follows: $270\;\mathrm{cm}\;\left(h\right)\times 50\;\mathrm{cm}\;\left(w\right)=1.35\;m^{2}\times 2\;\mathrm{faces}=2.70\;m^{2}$. The pixel coordinates shown in Figure 10(middle) are those of the mask generated during the instance segmentation process, and are used to calculate the real-world reconstruction of column Figure 10(right). The calculated surface area of the column is A=2.5104m${}^{2}$. The area of the front face (Figure 11(middle)) is A=1.1898m${}^{2}$, and the area of the side face is A=1.32055m${}^{2}$ (Figure 11(right)).

The percentage error in this case, i.e., the ABS ((2.5104 − 2.7)/2.7) × 100, is 7.022%.

**Figure 12:**
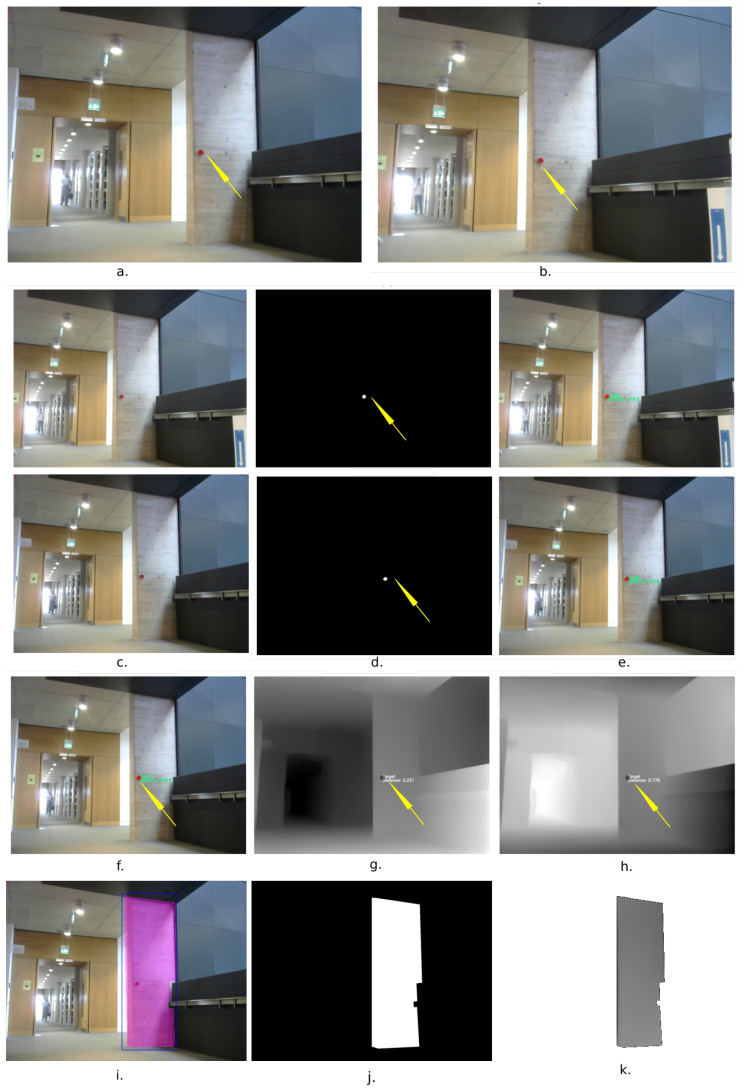
Experiment 2: estimation of the area of a concrete wall: in the first row scene capture (**a**,**b**), in the second are third row depth estimation to target in the left and right frames respectively (**c**–**e**), in the fourth row, the depth map (**g**), and corresponding inverse (**h**), using the left frame (**f**). In the last row, object segmentation (**i**), masking (**j**), and object extraction (**k**).

In the second experiment, the same stereo camera system was used with the baseline (b=25 cm) to capture the left and right frames of the scene containing a section of concrete wall as depicted in Figure 12a,b. The stereo camera system was placed at a distance of 5.6 m away from the wall section, with the target placed at the same level as the stereo system, where it is visible by both cameras (Figure 12a,b).

In this experiment, the calculated depth $Z_{xt,yt}$ to the centre of the target was 5.59 m, corresponding to $R_{xt,yt}=0.779$. Therefore, the scalar *S* in this case is:

(9) $$S=\frac{559}{0.779}=717.586\;\mathrm{cm}/\mathrm{px}.$$

Similarly, the inverted depth map shown in Figure 12h was multiplied by the scalar *S* to generate a dense map with absolute depths; it then passed the left frame through the trained module for object detection and instance segmentation to extract the concrete wall section, as shown in Figure 12i. The output mask, which is shown in Figure 12j, corresponding to the detected object, i.e., the concrete wall, is projected on the dense map with the absolute depth values, which were generated in the respective step of the first experiment to extract only those segmented pixels related to the concrete wall. Finally, the code in Algorithm 1 was applied to split the vertices belonging to the boundary (BV); we used Green’s theorem to estimate the area of the concrete wall.

**Figure 13:**
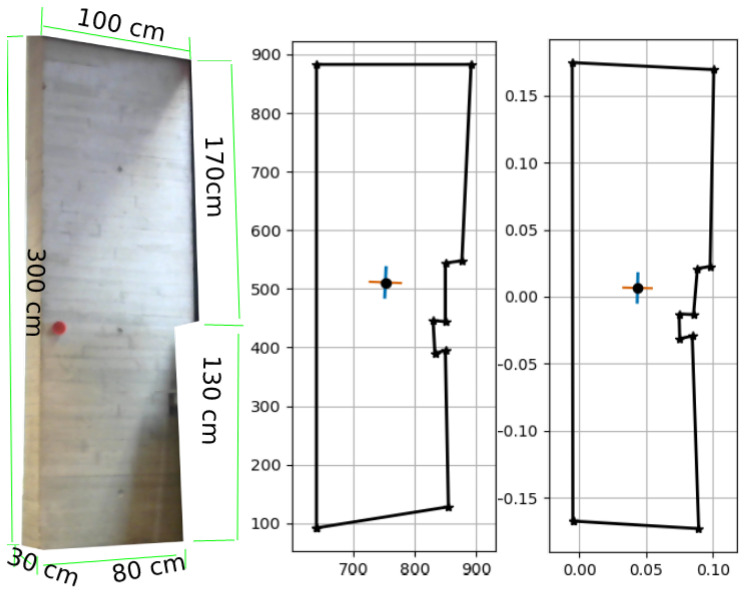
Real-world reconstruction of the concrete wall: the **left** is the total surface area, the **middle** is the front face area, and the **right** is the side face area.

The actual surface area of the wall section is slightly more complex, it is the sum of the bottom half, the upper half, and the side face, as shown in Figure 13(left): (130cm × 80cm)+(170cm × 100cm)+(30cm × 300cm)=36,400cm${}^{2}$ or 3.64m${}^{2}$. The pixel coordinates shown in Figure 13(middle) refer to the mask generated during the instance segmentation process and are used to calculate the real-world reconstruction of the column Figure 13(right). The calculated surface area of the column is A=3.3143m${}^{2}$. The percentage error in this case, i.e., the ABS ((3.3143 − 3.640)/3.640), × 100 is 8.947%.

The side face in this reconstruction is undetected; therefore, it is hard to estimate its contribution to the total estimated area.

### Limitations

Inferring the depth from a two-dimensional image is an extremely ill-posed problem. Errors may arise from many sources, but most importantly, the key contribution is attributed to the small scale at which the calculations are performed; i.e., the pixel scale, which will eventually be transformed to the real-world scale; i.e., meters, cm, feet, or inches.

The first error source comes from the camera calibration, which is the process of estimating intrinsic and/or extrinsic parameters of the camera. During this process, the focal length of the camera in pixels was estimated, which is the distance between the lens and the image sensor when the subject is in focus, and the principal point of the camera, which is the point on the image plane onto which the perspective centre is projected. There are other intrinsic parameters that contribute to errors, such as the skewness and the distortion of the lens, but in most cases, these factors are negligible.

The second error source comes from calculating the disparity from the horizontal displacement between the left and right frames. Regardless of the approach used to estimate the disparity, the main concept is to find the same pixel in both the left and right frames and calculate the difference between the *x*-components of that pixel.

There is another potential error that arises during the identification of the object of interest in a scene. With instance segmentation, a mask was used to select every pixel that is related to the (whole) object. Therefore, if the mask is poorly generated, this may lead to incorrect calculations. Finally, there is the well-established problem of estimating the absolute depths of each and every pixel in the scene. Whilst CNN-based methods are well-known to be able to generate relatively dense depth maps, they are very hard to train on a specific task. Sparse depth maps, on the other hand, are rough and are not suitable for geometric estimations that require a level of precision.

## 5. Conclusions

The proposed pipeline offers a fully automated computer vision-based method for measuring as-built elements of built assets.

The novelty of this work is attributed to the use of a single image and a single target to develop a fully automated computer vision-based method for measuring any given object.

Stereo camera techniques were used for data acquisition and deducing depth information. Machine learning, object detection, and instance segmentation techniques were also utilised to isolate the as-built element of interest and to process the geometric information of these elements. Finally, the principles of Green’s theorem were applied to estimate the size of the object(s). To demonstrate the results, a neural network was trained to detect and segment concrete walls and columns. A red disk target was placed in the field of view and we used a calibrated stereo camera system to capture the scene. A depth map was generated for this scene and the distance to the target was also calculated using triangulation methods. This information was then used to calculate the real-world dimensions of the object, which was then used to estimate the surface area. Limitations to the approach can arise during the camera calibration process and from calculating the disparity displacement between the left and right frames. Errors may also arise due to incorrect identification and segmentation of the object of interest, which may result in a poorly generated mask, which could lead to incorrect area calculations. The proposed pipeline was applied and tested on as-built elements within a university campus. However, we intend to further extend this work and examine the feasibility, scale-up, and practicality of the proposed fully automated CV-based method on real-life construction sites.

## Abbreviations

The following abbreviations are used in this manuscript:

3D	three-dimensional
AI	artificial intelligence
BIM	building information modelling
CV	computer vision
CNN	convolutional neural network
DLT	direct linear transformation
UAV	unmanned aerial vehicle
